# Efficient Treatment of Experimental Cerebral Malaria by an Artemisone-SMEDDS System: Impact of Application Route and Dosing Frequency

**DOI:** 10.1128/AAC.02106-20

**Published:** 2021-03-18

**Authors:** Johanna Zech, Nadeen Salaymeh, Nicholas H. Hunt, Karsten Mäder, Jacob Golenser

**Affiliations:** a Institute of Pharmacy, Martin Luther University Halle-Wittenberg, Halle (Saale), Germany; b Department of Microbiology and Molecular Genetics, The Kuvin Centre for the Study of Infectious and Tropical Diseases, The Hebrew University of Jerusalem, Jerusalem, Israel; c Discipline of Pathology, School of Medical Sciences, Faculty of Medicine and Health, The University of Sydney, NSW, Australia

**Keywords:** artemisone, malaria, SMEDDS, microemulsion, drug delivery, self-emulsifying, oral, nasal, intraperitoneal

## Abstract

Artemisone (ART) has been successfully tested *in vitro* and in animal models against several diseases. However, its poor aqueous solubility and limited chemical stability are serious challenges. We developed a self-microemulsifying drug delivery system (SMEDDS) that overcomes these limitations. Here, we demonstrate the efficacy of this formulation against experimental cerebral malaria in mice and the impact of its administration using different routes (gavage, intranasal delivery, and parenteral injections) and frequency on the efficacy of the treatment. The minimal effective daily oral dose was 20 mg/kg. We found that splitting a dose of 20 mg/kg ART given every 24 h, by administering two doses of 10 mg/kg each every 12 h, was highly effective and gave far superior results compared to 20 mg/kg once daily. We obtained the best results with nasal treatment; oral treatment was ranked second, and the least effective route of administration was intraperitoneal injection. A complete cure of experimental cerebral malaria could be achieved through choosing the optimal route of application, dose, and dosing interval. Altogether, the developed formulation combines easy manufacturing with high stability and could be a successful and very versatile carrier for the delivery of ART in the treatment of human severe malaria.

## Abstract (precis)

Artemisone (ART) has been successfully tested *in vitro* and in animal models against several diseases. However, its poor aqueous solubility and limited chemical stability are serious challenges.

## INTRODUCTION

Artemisinin derivatives are first-line antimalarial drugs (1). However, resistance to all commercial artemisinins, including their combinations with other antiplasmodial drugs, has been reported (2, 3). Artemisone (ART), a relatively new artemisinin derivative (4), was previously examined for plasmodial growth inhibition *in vitro* (5) and in mouse models of malaria and proved superior to commercially available artemisinin derivatives. ART has higher activity *in vivo* and *in vitro* compared to artesunate, is not metabolized to the neurotoxic dihydroartemisinin, unlike the other artemisinins, and is not exposed to severe first-pass metabolism (6). Consequently, it has a longer half-life (*t*_1/2_) *in vivo* and is about 3 to 5 times more active than artesunate, *in vitro* and *in vivo* (4, 7). ART toxicity has not been extensively evaluated *in vivo*, but we have not seen any immediate effects after using our formulation in the current and previous experiments (8). *In vitro* studies revealed a decreased toxicity compared to artesunate (9). Resistant plasmodia are more sensitive to ART than the currently used artemisinins (10–12).

*In vivo*, ART has been injected after solubilization in dimethyl sulfoxide (DMSO) (because of its poor aqueous solubility) (7) or inserted subcutaneously in solid or pasty polymers for slow release (13, 14) thereby necessitating a chirurgic intervention. Recently, we developed a new formulation for ART that overcomes the limitation of the lipophilic drug by dissolution in a self-microemulsifying drug delivery system (SMEDDS)/microemulsion (ME) (8). Its delivery is simple and may solve problems related to compliance. Oral administration of this formulation by gavage has been found very efficient in mice infected with Schistosoma mansoni (8).

It has been speculated that artemisinins are active against both schistosomes and plasmodia through the heme-initiated formation of free radicals (15). Consequently, it is the goal of this study to evaluate the efficacy of the ART-SMEDDS/ME formulation in a mouse model of cerebral malaria. We aimed to explore the impact of different administration routes (oral gavage, intranasal delivery, or parenteral injections) and frequency on the efficacy of the treatment.

The choice of an appropriate delivery system is crucial for the efficacy of treatment with any drug. ART is a biopharmaceutical classification system (BCS) class II drug with high permeability and low solubility (*M*_w_, 401.5 g/mol; aqueous solubility, 89 mg/liter; log *P*, 2.49) (6). For oral administration, a suitable delivery system must increase dissolution kinetics to enhance bioavailability (16). Likewise, Steyn et al. report that absorption of dissolved ART from their Pheroid delivery system in a mouse is almost 5-fold higher than that from a drug suspension (17). Furthermore, for the treatment of schistosomiasis in mice, we found that 40 mg/kg per dose was sufficient if ART was dissolved in our SMEDDS (8), while to obtain similar results with a suspension required 400 mg/kg of the drug (18).

A variety of complex delivery systems have been proposed for a broad range of routes of administration to overcome the low solubility and relatively poor stability of ART in an aqueous environment (Table 1).

**TABLE 1:** Published drug delivery systems for artemisone

Drug delivery system	Route of administration	Suggested application	Reference
Nanoemulsion	Topical	Cutaneous tuberculosis	86
		*In vitro*	
Nano-vesicular formulations (liposomes, transferosomes, niosomes, Pheroid system)	Topical	Cutaneous tuberculosis	87
		*In vitro*	
	Topical	Melanoma	88, 89
		Melanoma	
		*In vitro*	
	Oral	Malaria	17
Solid lipid nanoparticles	Topical	Melanoma	88, 89
		Melanoma	
		*In vitro*	
Solid lipid microparticles	Oral	Malaria	25
SMEDDS/microemulsion	Oral	Schistosomiasis	8
		In mice	
Solid polymer implants	Subcutaneous implantation	Malaria	13
		In mice	
	Subcutaneous implantation	Schistosomiasis	18
		In mice	
Injectable pasty polymer implants (poly [sebacic acid-ricinoleic acid] gel)	Subcutaneous injection	Malaria	14
		In mice	
	Subcutaneous injection	Schistosomiasis	18
		In mice	
Lipid matrix tablet	Oral	Malaria	90
Electrospun nanofibers	Infusion (*ex vivo* extraction)	Malaria	91
Macro-porous polymeric sponges	Implantation/infusion (*ex vivo* extraction)	Malaria	92

Most of the methods that are presented (Table 1) were not examined for their biological activity or were determined only *in vitro*. A few successful methods that were examined in mice are associated with a surgical procedure. The relevance of murine models to human malaria has been extensively studied and confirmed (19–21).

An effective therapy does not only depend on a formulation suitable for the drug but must also ensure applicability under the conditions in which treatment is delivered. Today, malaria is particularly prevalent in remote, rural areas where adequate health care may be hard to access. Hence, prereferral treatment can be vital for patient survival, as rapid progression and deterioration are common in cases of severe malaria (22). While for children under the age of six the WHO recommends the use of rectal artesunate, only parenteral administration is available for other age groups as prereferral medication (23, 24). Here, an oral or—in the case of coma or vomiting—intranasal formulation, easily administered without special skills or injection or infusion equipment, could provide immediate first access to antimalarial treatment. Additionally, the storage stability and cost of production of such a drug delivery system have to be considered to make it applicable in the poorly resourced regions where malaria is most common.

In addition to poor and variable absorption after oral administration, chemical stability is a challenge for ART and other artemisinins. Exposure to buffer solutions causes a degradation of 25% (pH 7.4) or 80% (pH 5) within 1 month (25). With the exception of our SMEDDS, all oral ART formulations tested *in vivo* that have been published are aqueous dispersions. Unfortunately, no stability data have been reported for these formulations. With the water-free SMEDDS, and even with a ME formed from SMEDDS and 50% water, we achieved good ART stability even at high ambient temperatures (at least 3 months, 30°C) (8).

Based on these considerations, we examined in an animal model our ART SMEDDS/ME formulation as a potential simple, first-response drug delivery system for severe or cerebral malaria. Microemulsions are thermodynamically stable, isotropic systems usually comprising an oil/lipophilic component, surfactant, cosurfactant and an aqueous phase with possibly a cosolvent (26, 27). If formulated as a water-free preconcentrate, they are classed as SMEDDS, which spontaneously form a ME when brought into contact with aqueous liquids, e.g., with the gastric or intestinal fluids. Liquid SMEDDS can be produced by simple mixing. Advantages include inexpensive manufacturing, desirable storage stability, different routes of application, and facile administration (28). They are thermodynamically stable and avoid—in contrast to thermodynamically unstable dispersions (e.g., emulsions, suspensions, and liposomes)—the need to control the particle size.

Consistent and fast drug absorption, usually reaching maximum concentration of drug in serum (*C*_max_) in less than a few hours, is observed for most drugs orally administered in SMEDDS/ME (29). Such pharmacokinetics are especially appropriate for ART because artemisinins are usually applied as fast-acting, first-response drugs to rapidly reduce the parasite burden. Oral SMEDDS are already marketed for prescription drugs (30, 31). ME and SMEDDS have been proposed as delivery systems for other artemisinin derivatives (32–36), but to our knowledge, apart from our formulation, no such system for ART has been developed so far.

In the case of cerebral malaria, the possibility of intranasal administration of ME, utilizing the proposed nose-to-brain transport system (37), could also facilitate direct targeting of plasmodia residing in the small vessels of the CNS. Efficacy of intranasal dihydroartemisinin and artesunate against cerebral malaria has already been shown in mice (38, 39).

Besides the drug delivery system, an appropriate dosing scheme is essential for adequate treatment. For artemisinin-based malaria therapy, the length of the interval during which a minimum parasiticidal drug concentration is maintained crucial for efficacy (40, 41). Although this correlation has sometimes been criticized as being oversimplified (42, 43), it has been proposed based on pharmacokinetic modeling that the combined periods of exposure to parasiticidal concentrations of the drug can determine the success of treatment. Prolonged killing times could even help to overcome slow parasite clearance and partial artemisinin resistance emerging in Southeast Asia, caused by mutations in the PfKelch13 propeller domain of Plasmodium falciparum (40, 44–46). This probability is of extreme importance, as artemisinins and artemisinin-based combination therapies are still the WHO-recommended first-line treatment for uncomplicated as well as severe malaria (1). Increasing or even preserving the potential of the most effective class of antimalarial drugs available today by simple changes to dosing regimens seems particularly attractive because it avoids the high risks and costs of developing new compounds and can be put into clinical practice within a short time with moderate development costs.

Based on the aspects mentioned above, and encouraged by the successful treatment of experimental schistosomiasis with the ART SMEDDS/ME (9), we evaluated the effect of this formulation on experimental cerebral malaria in mice. In particular, we studied those parameters that could give insight into the pharmacokinetics of the system. Curative experiments were compared with drug serum levels and further investigated the impact of routes of administration (oral, intranasal, or parenteral) and dosing intervals on the therapeutic outcome.

## RESULTS AND DISCUSSION

### Drug delivery system.

The formulations used in this work were designed for both oral and intranasal administration by careful selection of the employed excipients. The low viscosity (10 millipascal-seconds [mPas]) of the SMEDDS-20 formulation (composed of 20% [wt/wt] SMEDDS-100 and 80% [wt/wt] PBS) is well suited for oral application via a thin gavage needle (20 gauge) and was therefore selected for administration via that route. SMEDDS-50 only comprises 50% phosphate-buffered saline (PBS) and therefore is considerably more viscous (118 mPas) than SMEDDS-20. However, increased viscosity is expected to prolong the residence time in the nasal cavity (47). Furthermore, only small volumes can be applied nasally, and a higher drug load is possible in SMEDDS-50 compared to SMEDDS-20 (8). Its neutral pH of around 6.5 is close to the pH in the nasal mucus (48), ensuring good tolerability and drug stability and a very moderate risk of precipitation of both drug and excipients at the ME-mucus interface.

The high concentration of surfactant and the surface activity of ME have given rise to the discussion and study of their local tolerability (48–51), e.g., for intranasal treatment. Diverse results were observed, depending on the nature and ratio of the excipients used, duration, and frequency of exposure. Though long-term toxicity cannot be ruled out for our formulation, no specific immediate signs of discomfort were observed in the animals treated orally, intranasally, or via intraperitoneal (i.p.) injections.

### Hemolysis.

Drug-free SMEDDS-100 and SMEDDS-100 containing 5% (wt/wt) ART were tested for their capacity to cause hemolysis. The formulations were assayed in concentrations of up to 1 mg SMEDDS/ml final erythrocyte suspensions in PBS and had no hemolytic activity (measured hemolysis, <0.1%). To further confirm that neither high drug concentrations nor the large amount of the surfactant in the SMEDDS could cause hemolysis, 50 μg/ml ART (dissolved in DMSO) and 1 mg/ml Kolliphor HS15 also were examined individually. No hemolytic activity was found. Triton X 1% (wt/vol) in PBS (positive control) lysed all erythrocytes, while no hemolysis was observed for physiological saline.

### *In vivo* experiments.

The mouse model used in the experiments results in the death of about 95% of the untreated C57BL/6 mice from a pathology closely resembling cerebral malaria in humans (52). Deaths occurred on days 6 to 9 postinoculation (p.i.) with 5 × 10^4^ erythrocytes parasitized by Plasmodium berghei ANKA, in compliance with the protocol for the model (7). We observed impairment of the blood-brain barrier by penetration of Evan’s blue into the brain parenchyma of infected mice and early death at relatively low parasitemia when mice were not anemic, equivalent to what is described in reference 14. A close observation of the same model revealed similar courses of parasitemia and mortality and activation of brain microglia and astrocytes, as well as typical intracerebral hemorrhages (53).

### Artemisone serum concentrations.

Serum concentrations of ART were measured via liquid chromatography-mass spectrometry (LC-MS) in serum collected from mice 2 and 8 h after gavage of either 40 mg/kg ART in SMEDDS-20 or placebo (Table 2). As expected, no drug was found in the serum of the placebo-treated mice. After 8 h, ART could still be detected in the serum samples of all three animals in that group, but in two of those mice, serum levels were below the lower limit of quantification of 180 ng/ml. Nine hundred to 2,300 ng/ml ART was present after 2 h, 10 times the maximum concentration found in the first human tolerability studies (140.2 ng/ml) (4) and a thousand times higher than the *in vitro* 50% effective concentration (EC_50_) against P. falciparum (1 to 2 ng/ml), determined for our formulation and for ART dissolved in DMSO (8, 10).

**TABLE 2:** ART serum concentrations found in mice (*n* = 3 per time point) 2 and 8 h after treatment with either placebo or 40 mg/kg body weight ART in SMEDDS-20 by gavage

Time postadministration	Administered formulation	ART serum (μg/ml)^*a*^
2 h	Placebo ART 40 mg/kg	0 |0 |0 0.9 |2.3 |1.3
8 h	ART 40 mg/kg	0.6 |< LLQ |< LLQ

a LLQ, lower limit of quantification = 0.18 μg/ml. Vertical lines separate results of individual mice.

The high bioavailability of ART within 2 h after dosing and its rapid elimination by 8 h are in accordance with what is generally observed for a variety of drugs orally administered in ME and SMEDDS formulations, such as Neoral and Norvir (29–31, 54).

Based on a *t*_1/2_ of only 2.8 h found in humans (4), the results suggest that after 2 h, ART uptake is mostly complete: for the average of 1.5 μg/ml ART found after 2 h, about 0.34 μg/ml would remain at 8 h if no more drug absorption occurred.

The observed variations in serum concentrations can be attributed to the mice not being fasted before dosing. The feeding before or after gavage could affect the delivery of ART, through either pH, gastrointestinal motility and stomach passage times, digestive enzymes, or interaction with food components. We did not apply fasting before or after gavage because fasting by itself would affect the outcome of the disease (55).

### Prophylactic treatment.

The ED_50_ for P. falciparum of ART in our SMEDDS was found to be 1 to 2 ng/ml *in vitro* (8). Hence, it is reasonable to expect *in vivo* effects even at serum concentrations below the lower limit of detection (LLD) of our LC-MS method (60 ng/ml). Therefore, we used prophylactic treatment, prior to parasite inoculation, as an *in vivo* bioassay to gain additional information on the pharmacokinetics of the formulations and their effects *in vivo*.

To rule out any antimalarial effect of the plain ME *in vivo*, mice were treated with drug-free formulations, via gavage or intranasally, once a day on days 3 to 5 p.i. All animals exhibited characteristic clinical signs and had to be sacrificed or died of cerebral malaria less than 24 h before or after the untreated control mice (Table 3). The parasitemia in all mice was below 15%, confirming that the vehicle did not alter the course of the infection. Hence, there was no need to include additional placebo groups in further treatment studies.

**TABLE 3:** Day of death from severe malaria of mice treated with drug-free SMEDDS (parasitemia in all mice were below 15% throughout the experiment)

Treatment group	Administered formulation	Day of death (day p.i.)^*a*^
Control		8 |8 |8 |8 |8 |9
Oral (gavage)	SMEDDS-20 200 μl	8 |8 |8 |9
Intranasal	SMEDDS-50 20 μl	7 |7 |7 |7 |7

a Vertical lines separate results of individual mice.

The prophylactic treatment with ART-loaded SMEDDS was performed in mice that were administered 40 mg/kg ART via gavage once, at a specific time point before inoculation of malaria-infected erythrocytes. Any changes in the course of the disease can thus be attributed to the amount of drug present at the time of inoculation. Treatment 2 or 8 h before infection delayed the appearance of parasites in the bloodstream by 24 to 48 h and resulted in a shift of the disease from cerebral to anemic malaria in 3/5 and 2/4 animals, respectively (data not shown).

**FIG 1:**
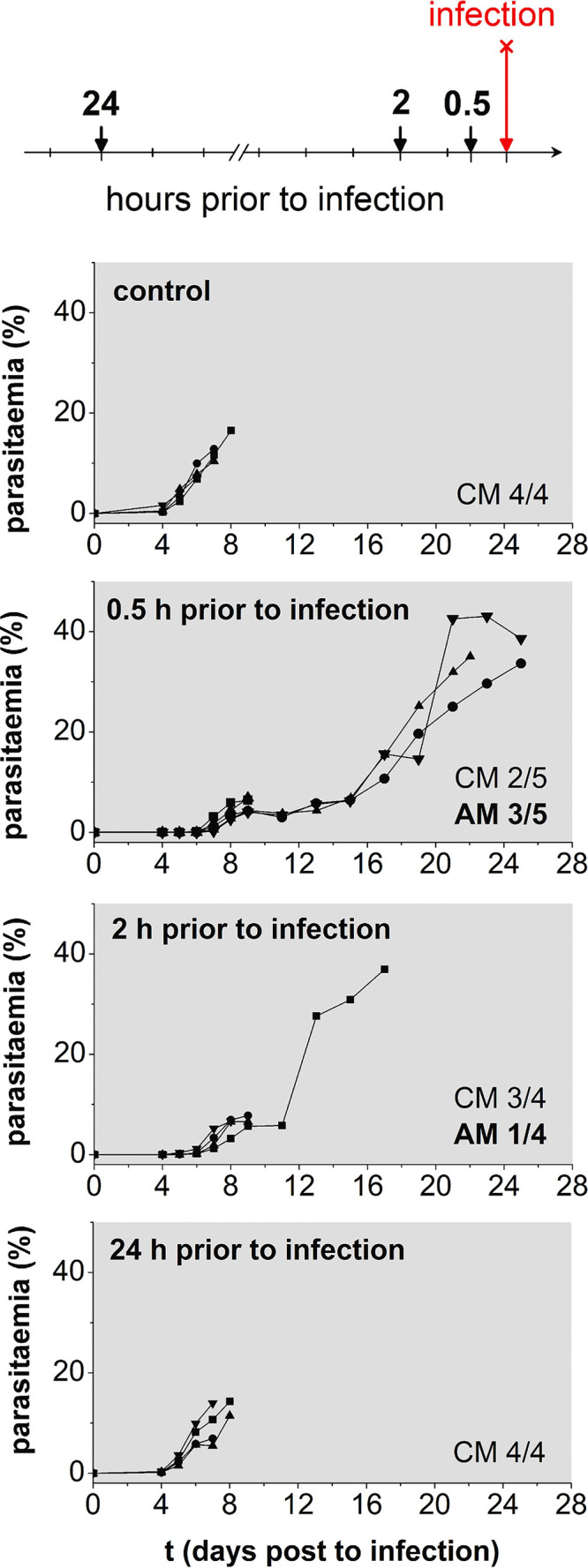
Prophylactic treatment. Parasitemias of mice treated once with ART at 40 mg/kg bodyweight in 200 μl SMEDDS-20 via gavage 0.5, 2, or 24 h prior to infection with P. berghei ANKA. The number of animals succumbing to either cerebral malaria (CM) or anemic malaria (AM) is given for each group.

The experiment was repeated, including 0.5, 2, and 24 h before infection, to cover a more comprehensive time range in which ART could be present in the serum (Fig. 1).

Treatment 24 h prior to inoculation led to drug concentrations that were too low to change the course of the infection compared to the untreated control group, as all animals in both groups died of cerebral malaria on day 8 p.i. The appearance of parasites in the bloodstream was delayed by 48 h if mice were given ART 0.5 or 2 h before infection. A shift from cerebral to anemic malaria occurred in 3/5 and 1/4 mice, respectively. The difference between the number of days that death was delayed in the anemic mice, in the two experiments, is not statistically significant (*t* test, *P* > 0.05). Nevertheless, more animals experienced a shift to anemic malaria if treated at time points closer to infection. For other small molecules in microemulsions, *C*_max_ was reached as soon as 30 to 60 min after gavage (56, 57). With similar kinetics for our experiment, the time during which the plasmodia are subjected to the minimum parasiticidal concentration (MPC) could be considerably longer for ART given only 30 min prior to infection. This prolonged “effective time” could, in consequence, lead to a greater reduction of the parasite load.

The serum ART concentrations and their resulting effects suggest a fast drug uptake into the blood within an hour after dosing, followed by a first-order, *t*_1/2_-controlled elimination of ART. Overall, we conclude that enough drug remains after 8 h for antiplasmodial activity, but not after 24 h.

### Dose response.

The dose response was confirmed for orally administered ART in SMEDDS-20 in a mouse model of cerebral malaria. For P. berghei ANKA infection in mice, a 50% effective dose (ED_90_) of 11.67 mg/kg has been described after oral administration of an aqueous ART suspension on days 0 to 3 p.i. (10). Considering the increased bioavailability of most small, lipophilic drugs if administered in a ME, we anticipated a more pronounced effect for our formulation.

**FIG 2:**
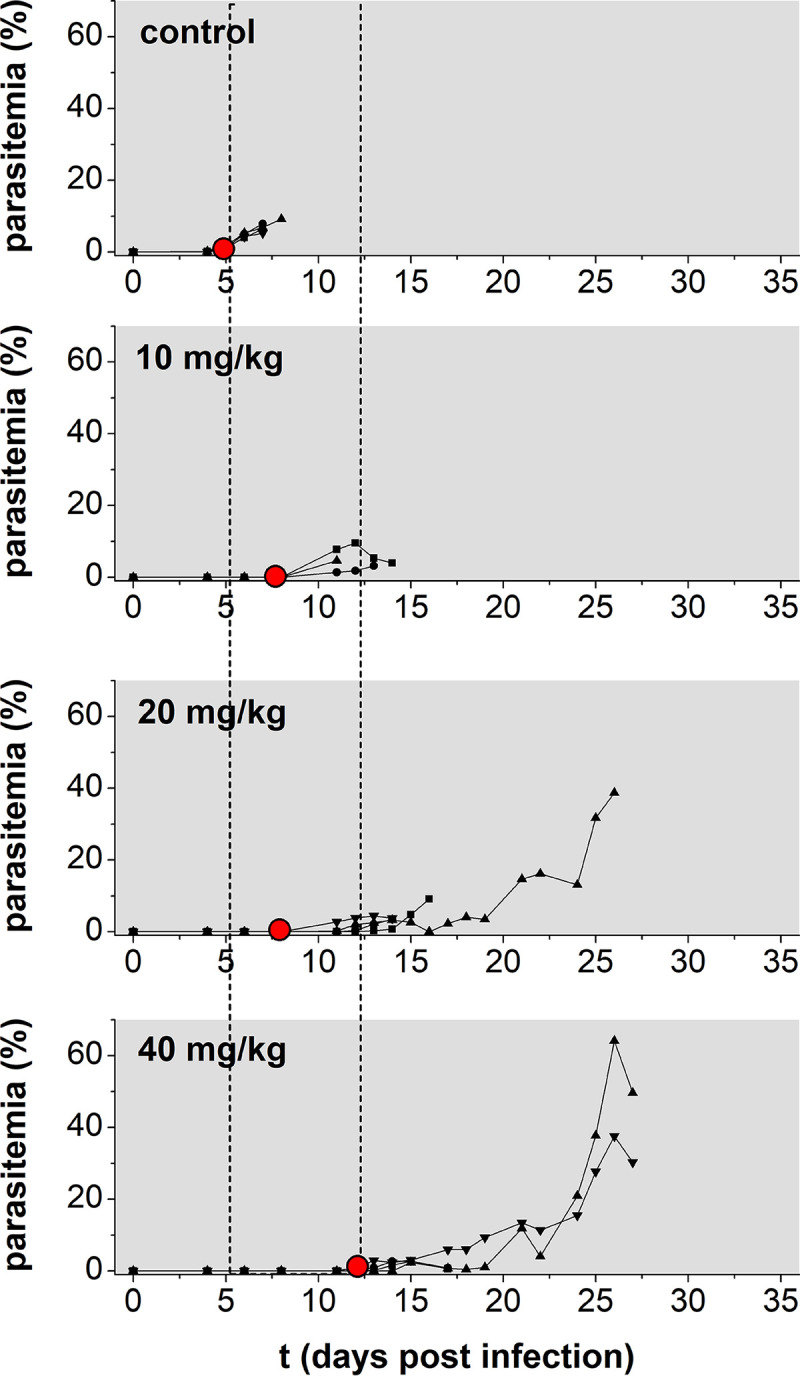
Dose-response study in infected mice. Parasitemia in mice that were treated with 10 to 40 mg ART/kg bodyweight via gavage every 24 h on day 3 to 5 p.i.; the red dot indicates the earliest detection of parasites in the bloodstream.

Animals were therefore treated with 10, 20, or 40 mg/kg ART in SMEDDS-20 once a day on days 3 to 5 p.i. No signs of toxicity were observed, but mice treated with the 40-mg/kg dose appeared more alert and hyperactive during a short period after treatment. Clear dose dependence was found for the delay of parasite detection in the blood and for the survival of the animals. The 40-mg/kg dose delayed the appearance of detectable parasites in the peripheral blood by 1 week. While 10 mg/kg was not enough to shift the course of the infection from cerebral malaria to anemic malaria, such a shift occurred in mice that received 20 or 40 mg/kg, respectively (Fig. 2). The combined results of two separate experiments indicate a dose-dependent pattern of response (Table 4); while no animals survived if administered 10 mg/kg, giving 20 and 40 mg/kg ART led to infection-free survival rates of 20 and 30%, respectively. A shift from cerebral malaria to anemic malaria occurred in 4/10 animals in all the groups but was only observed in the second run for the 10 mg/kg dose. Such a shift would enable a thorough diagnosis and additional treatment of infected individuals. In view of the results, a daily dose of at least 20 mg/kg was used as the standard dose in further experiments.

**TABLE 4:** Dose-response study in infected mice^*a*^

Treatment group	CM	AM	Survived
Control (*n* = 9)	9		
10 mg ART/kg (*n* = 9)	5	4	
20 mg ART/kg (*n* = 10)	4	4	2
40 mg ART/kg (*n* = 10)	3	4	3

a The mice were treated with 10 to 40 mg ART/kg bodyweight by gavage every 24 h on days 3 to 5 p.i. Combined results of two identical experiments are presented as number of deaths due to cerebral malaria (CM) or anemic malaria (AM), and parasite-free survival.

The results imply that, similar to what has been described for artesunate in humans (58), there is a ceiling effect for ART if orally administered in the SMEDDS every 24 h at around 20 mg/kg. This indicated that a decrease in dosing interval rather than a higher drug dose might increase the efficacy of the treatment.

A chi-square test reveals an overall significant effect of the treatments (*P* = 0.036). There is an increase in survival parallel to increasing ART dose, and examination of the effects of combined anemic malaria (AM) and survival reveals increased significance (*P* = 0.013).

### Dosing interval.

**FIG 3:**
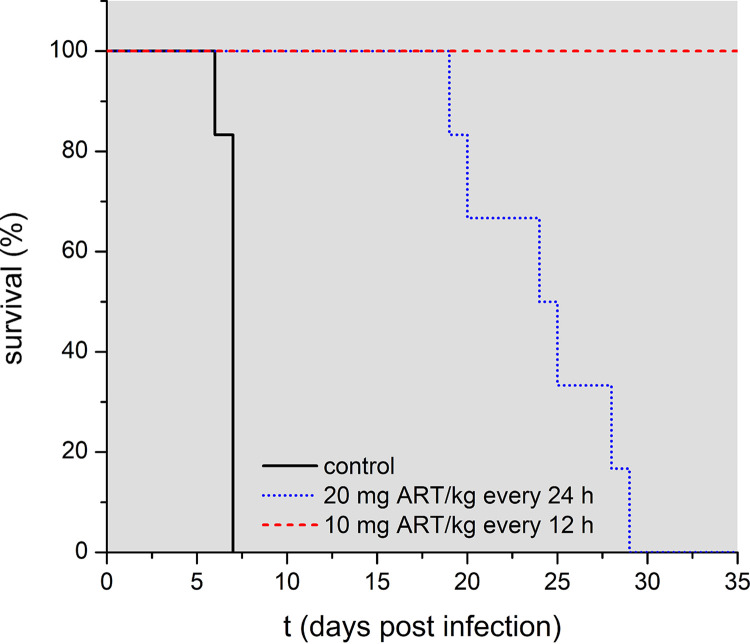
The effect of different dosing intervals on survival of mice treated by gavage on days 3 to 5 p.i. with 20 mg ART/kg bodyweight every 24 h in 200 μl SMEDDS-20 (*n* = 6) or 10 mg ART/kg every 12 h in 100 μl SMEDDS-20 (*n* = 5); control mice, *n* = 8. The significance of the influence of the treatment on the survival of the mice was tested using SPSS. Based on the log rank test data, the treatment regime had a highly significant influence on the survival (*P* < 0.001).

The influence of treatment frequency on the efficacy of artemisinin-based combination therapies (ACTs) for malaria patients has frequently been debated in recent years (40, 44, 58–60). We found that splitting a dose of 20 mg/kg ART given every 24 h into two doses of 10 mg/kg each, every 12 h, dramatically improved the outcome. While all mice that received 20 mg/kg in 200 μl SMEDDS-20 once daily died of anemic malaria, parasite-free survival was reached in all animals that were given a split dose of 10 mg/kg in 100 μl SMEDDS every 12 h (Fig. 3 and Table 5).

**TABLE 5:** Dosing interval study in infected mice^*a*^

Treatment group	CM	AM	Survived
Control (*n* = 8)	7	1	
10 mg ART/kg every 12 h (*n* = 7)			7
20 mg ART/ kg every 24 h (*n* = 10)		9	1

a Combined results of two identical experiments are presented. Mice were treated by gavage on days 3 to 5 p.i. with (i) 20 mg ART/kg body weight every 24 h in 200 μl SMEDDS-20 and (ii) 10 mg ART/kg every 12 h in 100 μl SMEDDS-20. Results are presented as the number of deaths due to cerebral malaria (CM) or anemic malaria (AM) and parasite-free survival.

In general, two parameters are essential for choosing the right dosing interval in malaria treatment. (i) The stage-specificity of the drug has to be considered in cases of infections that present synchronized cycles of parasite blood stages. The maximum efficacy of a drug can only be exploited if the stages most sensitive to the drug are present at the time it reaches concentrations above the minimum parasiticidal concentration (MPC) in the bloodstream (41, 60). The artemisinins are well known for their activity against very early ring stages of P. falciparum (41), though trophozoites and schizonts also have been reported to be sensitive (61, 62). However, artemisinins increasingly accumulate in parasitized erythrocytes in accordance with intracellular parasite development (63). In the Plasmodium berghei ANKA mouse model, parasites are not age-synchronized before infection, and sensitive parasites are most likely always present, though their percentage might vary. In any event, the Plasmodium berghei ANKA-C57BL/6 mouse combination is an appropriate model for human cerebral malaria (20).

(ii) The amount of time during which drug serum concentrations above the MPC are maintained determines the outcome of the treatment. If a twice-daily, half-dose regime leads to an increase in combined killing time per day (compared to the once-daily administration of the total dose), the total number of killed or damaged parasites is obviously larger, as explained by Kay et al. (40). This could be the case if the administered drug has a ceiling effect, something observed for artesunate (58) and for ART in our dose-dependence studies, where increasing the amount of ART from 20 to 40 mg/kg only slightly improved efficacy. Pharmacological modeling has predicted that by splitting the daily dose of artemisinins and doubling the treatment frequency, the killing time would almost be doubled, increasing parasite eradication and dramatically reducing treatment failure rates of P. falciparum infections in humans (40, 64). Unfortunately, *in vivo* studies in patients failed to support this theoretical calculation (58, 59, 65). Possible explanations for the differing results from these clinical studies—the influence of reduced ring-stage susceptibility due to resistance, as well as acquired immunity in the patients—have been discussed (60, 64). These factors are not relevant in our experimental system that uses naive mice and a Plasmodium berghei ANKA strain that is not drug resistant. Rather, Plasmodium berghei ANKA infections in mice induce a fulminant lethal disease, similar to human severe and cerebral malaria in nonimmune patients (52).

Many field studies use parasite clearance in the peripheral blood following treatment as the endpoint, a method that overlooks noncirculating parasites while considering dead but not yet cleared blood-stage plasmodia. When the number of patients succumbing to severe malaria is below 2%, a reliable statistic analysis based on mortality/parasite-free survival is difficult (59, 64, 65). In our experiments in mice, 20 mg/kg ART every 24 h did not save most animals but could shift the disease from cerebral malaria toward anemia that killed the animals about a week after the death of the untreated animals. A similar delay in patients might be crucial in saving lives by enabling proper diagnosis and treatment. However, the split dose delivered every 12 h led to superior efficacy, with 100% parasite-free survival. Caution is needed in comparing the results of treatment of people infected with malaria to laboratory results of treatment of experimental cerebral malaria: the severity of human infections is not necessarily known, and concomitant infections with other infectious agents are common. The immune status also is not well defined, especially toward malaria in areas of endemicity. The situations of resistance to artemisinins and even drug consumption (antimalarial and other drugs) are not defined. Obviously, in our preclinical study we avoided these factors by using naive mice and a drug-sensitive Plasmodium berghei ANKA strain. In human clinical studies, when ACT was used (58, 59, 65) the observed fast onset of parasite reduction was mainly due to the artemisinin component, whereas the survival outcome might be a result of the drug combination (64). Therefore, our experiments, with mice receiving only ART, specifically show the effect of altering the dosing regimen of this agent in the absence of the actions of other drugs.

Taken together, these factors could have contributed to our finding that the results of the dosing interval study precisely confirm the predicted effect of the pharmacological modeling, with a split dose of 10 mg/kg twice a day curing all mice in that treatment group (Fig. 3).

The prophylactic experiment (Fig. 1) implies that 24 h after administration of 20 mg/kg ART, an insufficient concentration of the drug remains in the blood to kill the injected parasites. As a consequence, in the one dose per day treatment regime, there is a time period during which no killing of parasites will occur. Considering that 8 h after drug delivery an effect of the drug was still found, it is possible that remaining drug concentrations of a 10 mg/kg dose might be high enough to still affect parasite viability until the administration of the next drug dose. This could be examined by further prophylactic studies. Thus, increasing the treatment frequency rather than elevating the administered dose may improve the outcome and also reduce the *C*_max_ and consequent drug-induced side effects and toxicity.

### Route of application.

**FIG 4:**
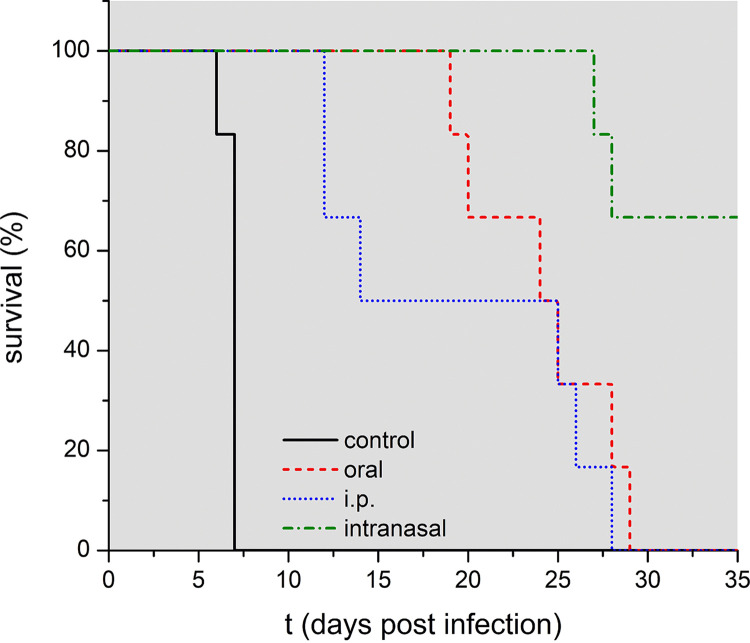
The effect of the route of application on mouse survival. Mice were treated with 20 mg ART/kg bodyweight on days 3 to 5 p.i. every 24 h by gavage (*n* = 6), i.p. injection (*n* = 6), and intranasal administration (*n* = 6); control mice, *n* = 8. A significant influence of the application route on the survival of the mice was tested using SPSS. Based on the log rank test data, the application route had a highly significant influence on the survival (*P*  <  0.001).

Three different routes of administration, oral, intranasal, and parenteral—were compared by administering 20 mg/kg ART every 24 h on days 3 to 5 p.i. (Fig. 4, Table 6). As a parenteral route, SMEDDS-20 was delivered via i.p. injections, a method that is more often applied to animals than to humans (38, 66).

**TABLE 6:** The effect of the route of application on infection outcome^*a*^

Treatment group	CM	AM	Survived
Control (*n* = 8)	7	1	
Oral (gavage) (*n* = 10)		9	1
Intranasal (*n* = 8)		4	4
i.p. (*n* = 10)	3	7	

a Combined results of two identical experiments are presented. Mice were treated with 20 mg ART/kg body weight every 24 h on days 3 to 5 p.i. by gavage/i.p. injection/intranasally. Results are given as deaths due to cerebral malaria (CM) or anemic malaria (AM) and parasite-free survival.

Administration of ART i.p. delayed death but still led to cerebral malaria in 30% of the animals. After oral treatment, 90% of the mice showed a shift to anemic malaria with consequent prolonged survival, and in one mouse, no parasites could be detected until the end of the experiment. Intranasal ART administration saved 50% of the mice, and anemic malaria occurred in the other half. The efficacy can be rated as i.p. < oral < nasal administration, and this order was confirmed by repeating the experiment (both experiments are summarized in Table 6).

The different outcomes for the studied routes of drug administration could possibly be explained by variations in (i) bioavailability, (ii) the time of drug uptake and maintenance period of MPC, and (iii) the tissues/compartments that are reached by ART.

Injections via the i.p. route, similar to i.v. administration, lead to 100% bioavailability of the given drug, and we expect a high *C*_max_ at a short time to maximum concentration of drug in serum (*T*_max_) (66). Consequently, neither a large total amount of available drug nor exceptionally high serum concentrations can be considered responsible for the more effective treatment provided by oral and intranasal dosing. Instead, they presumably are due to either prolonged drug uptake and consequently longer exposure of the parasite or delivery of the drug to different compartments, e.g., the brain.

The effect of the extended killing time has already been discussed, with more hours/day above the MPC resulting in the elimination of more parasites. It is essential to consider the length of individual drug pulses, during which drug concentrations above the MPC are reached. Longer durations of individual treatments were more effective in decreasing the number of parasites that survive initial exposure to artemisinins (41), due to the increased probability of exposure of parasite stages that are more sensitive to the drugs. A route of application for our SMEDDS that provides extended drug uptake could, therefore, considerably increase the effectiveness of ART.

After oral administration, passage along the gastrointestinal tract and interaction with food components may lengthen the drug uptake. For intranasal dosing, two mechanisms have been shown to prolong the duration of drug delivery; the nasal mucosa can act as a drug reservoir, suggesting continued drug release even after clearance of the formulation from the nasal cavity (67). In addition, the high viscosity of the SMEDDS is likely to considerably increase the time of residency of the ME on the nasal mucosa (47). In C57BL/6 mice, a mucociliary transport of 1.3 mm/min would remove low-viscosity aqueous solutions from the nasal cavity in less than 20 min (68), but a considerably longer time of residency and consequent drug uptake can be assumed for our formulation with a viscosity of 118 mPas (9). However, for other small-molecule drugs delivered intranasally in ME, no prolonged drug release could be observed when compared to oral administration (50, 51, 69, 70). Rather, an exceptionally fast drug uptake was found when comparing serum concentration time curves (51, 70–74). For a mirtazapine ME, a higher *C*_max_ and area under the inhibitory curve (AUC), but shorter *T*_max_ and *t*_1/2_ are reached if applied intranasally instead of orally (69).

Nasal administration has been studied as a method to directly target the brain via the uptake of the drug through the olfactory region of the nasal epithelium and branches of the trigeminal nerve (37). This nose-to-brain transport should be especially effective in rodents, considering their—compared to humans—large intranasal surface area, consisting of 50% olfactory epithelium (75). High drug concentrations in the brain following intranasal administration in ME have been reported for a variety of small-molecule drugs compared to the i.v. or oral routes (69–71, 73, 76). There is evidence for intravascular accumulation of parasitized erythrocytes in brain vessels in the mouse model and in human cerebral malaria (77). Consequently, targeting the brain would be an advantage, and enhanced treatment of parasites in the brain is, therefore, a possible explanation for the efficacy of intranasal administration of ART. A different explanation could be an increase of the apparent plasma half-life of ART, with the cerebrospinal fluid and brain tissue forming a drug reservoir, protecting ART from rapid metabolism and slowly releasing it into the plasma. A prolonged brain half-life can be found for other lipophilic small-molecule drugs after oral delivery (78, 79). But as little is known about the metabolism and excretion of ART from the central nervous system (CNS), this must remain speculative for now. For other artemisinin derivatives, prolonged brain and plasma concentrations could not be observed after intranasal administration. However, those experiments used nano lipid carriers (80) and aqueous solutions (39). The latter have been found to be inferior in intranasal drug delivery compared to ME (50, 70, 71, 76).

Despite the pronounced result of nasal delivery, often this method is not optimal for measuring biodistribution because the drug may reach various organs—the brain, the respiratory tract, and the intestine (37, 81). In addition, frequent use of this route may lead to mucosal damage. Therefore, we focused on gavage and consequently could more accurately estimate the availability of the drug in the blood, which is the crucial factor in parasite elimination. However, faced with the challenge of treating a potentially unconscious or vomiting severe malaria patient, intranasal administration might be a beneficial instrument to possess. Thus, the intranasal route was of interest to us and, hence, was included in this study.

Overall, both prolonged delivery with consequently extended potential killing duration as well as delivery to the brain (for intranasal treatment) are to be considered possible explanations for the increased efficacy of the oral and intranasal route in our experiments and for future applications.

### Late treatment.

Delayed treatment, on days 6 to 9 p.i. when mice were suffering from fulminant disease, was studied. Mice were treated every 12 h with 20 mg/kg drug intranasally or 40 mg/kg via gavage (Table 7). Parasitemia dropped in all mice, from 1 to 3% on day 6 postinfection, to below the detection limit within 48 h after the beginning of treatment. In the animals that died of severe malaria, parasites were absent for at least 10 days posttreatment, with deaths occurring between days 21 and 24 p.i. The effective late treatment with a high parasite reduction rate is common for artemisinins (43), and the consequent time window before recurrence of the plasmodia can be critical in clinical cases. It would allow the rescue of already severely infected patients and subsequent treatment with other, longer-acting antimalarial drugs.

**TABLE 7:** The effect of late treatment on infected mice dosed every 12 h on days 6 to 9 p.i. with either 20 mg ART/kg body weight intranasally or 40 mg ART/kg via gavage^*a*^

Treatment group	CM	AM	Survived
Control (*n* = 9)	8	1	
40 mg ART / kg oral (gavage) (*n* = 9)	3		6
20 mg ART/kg intranasal (*n* =10)	3		7

a Combined results of two experiments are shown as deaths due to cerebral malaria (AM) or anemic malaria (AM) and parasite-free survival.

### Conclusion.

Because complete cure of experimental cerebral malaria was achieved when using the optimal route of application, dose, and dosing interval, our formulation is altogether a promising, very versatile carrier for the delivery of ART in the treatment of severe or cerebral malaria.

## MATERIALS AND METHODS

Artemisone (ART) was kindly donated by Cipla (Mumbai, India) and was used for the experimental procedures that are included in this paper. Artemisone that had been donated by Richard K. Haynes (The North-West University, Potchefstroom, South Africa) was previously used for the development of the ART formulation (8). Polysorb ID 46 (isosorbide caprylocaprate diester) was a gift from Roquette, (Lestrem, France). Capmul MCM EP (glycerol monocaprylocaprate) was obtained from Abitec (Columbus, OH, USA). Kolliphor HS15 (polyoxyl 15 hydroxystearate), supplied by BASF (Ludwigshafen, Germany), was melted and homogenized before use to ensure a homogenous composition. Propylene glycol and indomethacin were purchased from Caesar & Lorentz GmbH (Hilden, Germany). All chemicals were of analytical grade. High-pressure liquid chromatography (HPLC) solvents were purchased as HPLC grade.

Sterile filtered mouse serum was purchased from Biowest (Nuaillé, France). Phosphate-buffered saline pH 7.4 (PBS) was manufactured by Biological Industries (Bet HaEmek, Israel). Double distilled water only was used in the experiments.

### Drug delivery system.

The SMEDDS composition is given in Table 8, and a detailed description of the formulation development and characteristics is found in reference 8. Briefly, the combination of polar lipid excipients forms a SMEDDS that provides high solubility (59 mg/g) and stability of ART in both the water-free SMEDDS itself and the corresponding microemulsions (ME) obtained by the addition of H_2_O or PBS. SMEDDS-50 and SMEDDS-20 formulations were prepared from a solution of ART in SMEDDS-100 (or drug-free SMEDSS-100 for placebo) immediately before administration. Specific amounts of PBS were added to give SMEDDS-50 or SMEDDS-20 (Table 8), and the ME was then sterile-filtered through a syringe filter.

**TABLE 8:**
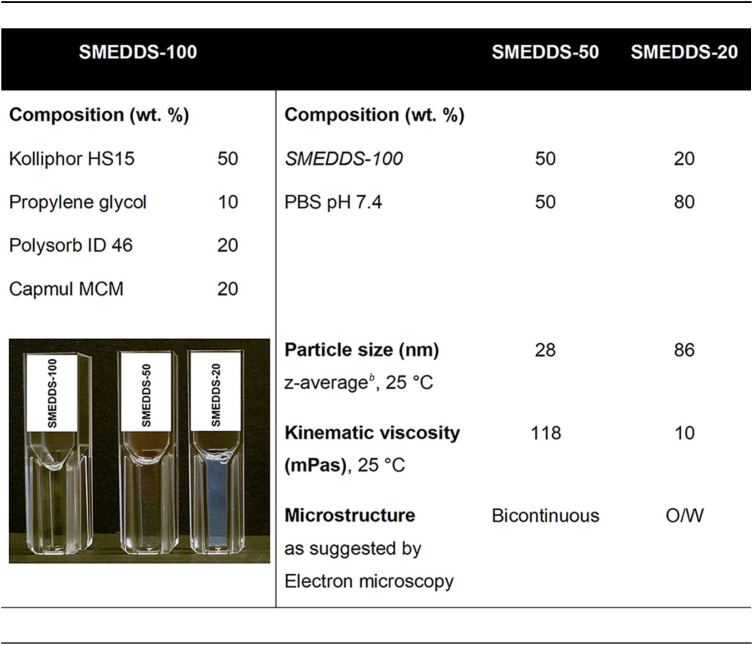
Composition and properties of the water-free SMEDDS-100 and the microemulsions SMEDDS-50 and SMEDDS-20 derived from it, as used in this work^*a*^

a The image demonstrates the transparency of the three formulations and the opalescence observed for SMEDDS-20.

b Dynamic light scattering, intensity diameter distribution.

### Hemolysis.

A suspension of 8 × 10^9^ human red blood cells/ml was prepared in PBS. The examined formulations were added in 500 μl to the equal volume of cell suspension. Triton X served as a positive control (known to cause total hemolysis), whereas saline (0.154 mol/liter NaCl) served as a negative control. The sample plates were incubated for 1 h at 37°C and then centrifuged, and the optical density of the supernatants was measured at a wavelength of 540 nm using a UV/Vis spectrometer.

### *In vivo* experiments.

**(i) Animals.** Experiments were carried out using C57BL/6 Ola-Hsd male mice (from Harlan, Israel) aged 7 to 8 weeks. For the determination of ART serum concentrations, 10-week-old male ICR Harlan-Sprague-Dawley (ICR) mice (from Harlan, Israel) were used instead (to obtain increased amounts of serum). Both mouse species succumb to severe malaria with indications of cerebral damage following infection with Plasmodium berghei ANKA (38).

The mice were housed in groups and maintained under standard conditions, a 12-h on/off light cycle and unlimited access to food and water. Experiments were performed in accordance with institutional guidelines for animal care following protocols approved by the Animal Ethical Care Committee of The Hebrew University of Jerusalem (protocol no. MD-12-1351; Golenser’s accreditation no. 12180).

**(ii) Infection of animals**. The strain of Plasmodium berghei ANKA (PbA MRA-311; CDC, Atlanta, GA) used in the experiments was maintained by serial transfer of parasitized erythrocytes (PE) from infected to naive mice. Experimental mice were infected by i.p. injections of 5 × 10^4^ PE from peripheral blood of infected passage mice, which caused fatal severe malaria with cerebral involvement on days 6 to 10 postinfection (p.i.) in at least 90% of the infected animals.

**(iii) Monitoring of infection.** Thin blood smears from tail blood were prepared every 2 to 3 days to monitor parasitemia in the infected mice. They were stained with Giemsa staining-solution, and the number of PE per 10,000 red blood cells was determined via light microscopy. Samples were also assessed for anemia and reticulocytosis.

**(iv) Severe malaria mouse model.** This work used C57BL/6 mice infected with Plasmodium berghei ANKA as a model of numerous aspects of severe malaria that manifest in humans. The model is well established and comprehensively described in references 82 and 83. Infection and pathology in our animals were confirmed to be in accordance with the severe malaria mouse model; untreated mice exhibited a variety of characteristic clinical signs as manifestations of the infection (ranging from a ruffled coat, hunching, and apathy to neurological symptoms such as convulsion and coma that are indicative of cerebral malaria). Dying mice were sacrificed 6 to 9 days p.i. The histology of hematoxylin and eosin-stained organs revealed mild but significant changes in tissues in the liver and spleen and few parasites in the brain (data not shown). Injections of Evans blue (i.v., 0.2 ml of 1% wt/vol in PBS/mouse) led to staining of the brain tissue of infected mice but not naive mice, demonstrating an effect of the infection to permeabilize the blood-brain barrier. Parasitemia levels in these mice were below 20%, and animals were categorized as suffering from cerebral malaria (CM).

Some animals in which the course of the disease was altered, e.g., by treatment, exhibited prolonged survival of up to 4 weeks. They showed gradually increasing signs of severe anemia (colorless eyes, cold skin, rapid weight loss, apathy), while blood smears revealed hyperparasitemia of 30 to 70% PE and reticulocytosis (>30% reticulocytes compared to 1 to 4% in noninfected mice). The hematocrit in three representative mice was found to be below 25% as measured by centrifugation of tail vein blood in heparinized capillaries. These animals were consequently classed as succumbing to anemic malaria (AM).

Mice that exhibited no recurrence of parasites in the tail blood smears after treatment until the end of the experiment, at least 28 days p.i. or 21 days posttreatment, were considered “survived.”

### Artemisone serum concentrations.

**(i) Dosing and sample collection.** Male ICR mice weighing about 50 g were fed via gavage with either 40 mg ART/kg body weight in 200 μl SMEDDS-20 or a 200 μl drug-free formulation. The animals were bled from the eye into MiniCollect tubes (0.8 ml; Z serum [Sep] clot activator; Greiner Bio-One, Kremsmünster, Austria) after 2 or 8 h (artemisone treated) and 2 h (placebo); *n* = 3 in all three groups.

After 60 min, cells were separated from the serum by centrifugation, and the serum was collected in vials of 100 μl each. Samples were then frozen and lyophilized before being stored at −80°C until LC-MS analysis.

**(ii) LC-MS analysis.** The lyophilized serum was reconstituted in 100 μl bi-distilled water. To each tube, 500 μl ethyl ether was added, and samples were centrifuged and stored at −20°C for 60 min. The supernatant was collected, and the solvent completely evaporated. The residue was then dissolved in a solution of 15 μg/ml indomethacin (internal standard) in ethanol and diluted as necessary before LC-MS analysis.

Artemisone was quantified using HPLC (Agilent/HP 1100 HPLC series, high-pressure pump, degasser, and autosampler; now Agilent Technologies, Inc., USA) with an LCQ Finnigan MAT mass spectrometer (Thermo/Finnigan, San Jose, CA, USA) as the detector. Experiments were performed on a Eurospher 100-5 C18 100 by 2-mm column (Knauer, Berlin, Germany). Data were processed with Xcalibur data acquisition software (Thermo Fisher Scientific, Waltham, MA, USA). The LC-MS method was developed and validated based on ICH (International Council for Harmonisation of Technical Requirements for Pharmaceuticals for Human Use) guidelines (84) (Table 9) (lower limit of detection [LLD], 60 ng/ml; lower limit of quantification [LLQ], 180 ng/ml; R^2^ > 0.993). As standards for validation and calibration, mouse serum spiked with different amounts of ART was lyophilized before being reconstituted and processed identically to the samples collected in the experiment. Indomethacin (15 μg/ml) served as internal standard (85).

**TABLE 9:** LC-MS parameters for quantitative analysis of artemisone in mouse serum

LC for:						
Mobile phase (V/V/V)		Flow rate	Sample injection vol		Column temp	Retention time
ACN	55	0.3 ml/min	10 μl		35°C	5.5 min Indomethacin
H_2_O	45					7.0 min ART
FA^*a*^	0.1					

**MS for:**						
**Ionization**				**Detection**		
Electrospray ionization (ESI)				Single-ion mode		
Positive-ion mode				*m/z* = 401.5 to 403.5 artemisone (M + 1)		
				*m/z* = 357.8 to 359.8 indomethacin (M + 1)		

a FA, formic acid.

### Treatment.

**(i) Drug administration.** Male, 8- to 9-week-old C57BL/6 mice were randomized into groups after infection. Untreated infected mice served as the control group in all the experiments. The animals were treated once or twice a day, days 3 to 5 or 6 to 9 (late rescue) p.i. (see “Experimental Design”). The microemulsions were administered either via i.p. injections or gavage or intranasally with a fine pipette. For intranasal administration, the mice were kept on their backs to allow the drug to reach the olfactory region/upper nasal cavity to promote possible nose-to-brain transport (37). Drugs were delivered in microemulsion volumes of 200 μl, 25 μl, and 100 μl for gavage, intranasal, and i.p. treatments, respectively. The PBS content was varied to obtain a viscosity that allowed administration and a sufficient solubility of ART (Tables 8 and 10). Drug concentration was adjusted as needed, between 10 and 40 mg/kg based on an average body weight of 25 g per mouse, and the respective drug-free microemulsions were administered as placebo. All drug doses given in this article are calculated as mg/kg bodyweight.

**TABLE 10:** Amounts of specific SMEDDS used in treatment experiments depending on the route of application

Route of application	Formulation	Vol (μl)^*a*^
Oral	SMEDDS-20	200
Nasal	SMEDDS-50	25
i.p.	SMEDDS-20	100

a Unless stated otherwise.

**(ii) Experimental design.** We focused our experiments on different aspects of pharmacokinetics, including the following parameters: (i) prophylactic treatment, *in vivo* bioassay complementary to ART serum concentrations, (ii) dose-response, (iii) dosing intervals (once/twice daily), (iv) impact of the route of application (oral/i.p./intranasal), and (v) late treatment (during days 6 to 9 p.i.)

The detailed design of each experiment is presented in the corresponding results section. All experiments were repeated once (except for one indicated variation).

### Statistical tests.

Chi-square tests were performed using GraphPad Prism.

All other statistical testing was done using IBM SPSS version 25. Kaplan-Meier survival tables and plots were generated using lifetime as time, death as event (surviving mice were censored), and treatment or application route as factor. Factor values were compared using an overall log rank (Mantel-Cox) test. The result is statistically significant when *P* ≤ 0.05 (α = 5.00%) and highly significant when *P* ≤ 0.001 (α = 0.10%).
